# A Quinol Anion as Catalytic Intermediate Coupling Proton Translocation With Electron Transfer in *E. coli* Respiratory Complex I

**DOI:** 10.3389/fchem.2021.672969

**Published:** 2021-05-07

**Authors:** Franziska Nuber, Luca Mérono, Sabrina Oppermann, Johannes Schimpf, Daniel Wohlwend, Thorsten Friedrich

**Affiliations:** Institut für Biochemie, Albert-Ludwigs-Universität, Freiburg, Germany

**Keywords:** NADH dehydrogenase, respiratory complex I, bioenergetics, proton-coupled electron transfer, quinone chemistry, *Escherichia coli*

## Abstract

Energy-converting NADH:ubiquinone oxidoreductase, respiratory complex I, plays a major role in cellular energy metabolism. It couples NADH oxidation and quinone reduction with the translocation of protons across the membrane, thus contributing to the protonmotive force. Complex I has an overall L-shaped structure with a peripheral arm catalyzing electron transfer and a membrane arm engaged in proton translocation. Although both reactions are arranged spatially separated, they are tightly coupled by a mechanism that is not fully understood. Using redox-difference UV-vis spectroscopy, an unknown redox component was identified in *Escherichia coli* complex I as reported earlier. A comparison of its spectrum with those obtained for different quinone species indicates features of a quinol anion. The re-oxidation kinetics of the quinol anion intermediate is significantly slower in the D213G^H^ variant that was previously shown to operate with disturbed quinone chemistry. Addition of the quinone-site inhibitor piericidin A led to strongly decreased absorption peaks in the difference spectrum. A hypothesis for a mechanism of proton-coupled electron transfer with the quinol anion as catalytically important intermediate in complex I is discussed.

## Introduction

The universal cellular energy currency adenosine triphosphate (ATP) is mainly produced by oxidative phosphorylation, a process that couples electron transfer with ATP synthesis. Electrons from reducing equivalents that are produced during catabolism are transferred in an exergonic reaction to oxygen to produce water. The released energy is used in an endergonic reaction to pump protons across the membrane, thus, establishing the protonmotive force (pmf). The pmf in turn drives ATP synthesis by ATP Synthase (Mitchell, 1961). Complex I contributes to the pmf by coupling NADH oxidation and quinone (Q) reduction with the translocation of protons across the membrane. The two reactions find a reflection in the two distinct structural parts, the peripheral arm, catalyzing the electron transfer reaction, and the membrane arm, conducting proton translocation (Hirst, 2013; Sazanov, 2015; Cabrera-Orefice et al., 2018; Parey et al., 2019). Mammalian complex I is a complicated machinery consisting of up to 45 subunits, while bacteria contain a simpler version comprising in general 14 subunits. These 14 subunits are conserved in all species containing an energy-converting NADH:Q oxidoreductase and they represent the core subunits required for catalytic activity of complex I (Gnandt et al., 2016). The structure of complex I from several organisms is known from either X-ray crystallography or single-particle electron cryo-microscopy (cryo-EM), at resolutions not exceeding 3.2 Å (Agip et al., 2019). Recent cryo-EM studies yielded structures of the mitochondrial complex from a yeast and from ovine at 2.7 and 2.3 Å, respectively (Grba and Hirst, 2020; Kampjut and Sazanov, 2020).

Still, coupling of the exergonic electron transfer reaction in the peripheral arm with the endergonic proton translocation in the membrane arm is not fully understood. NADH is oxidized at the tip of the peripheral arm and the electrons are transferred from a flavin mononucleotide (FMN), the primary electron acceptor, via a chain of iron-sulfur (Fe/S)-clusters to the Q reduction site in ~100 Å distance. Q binds in 12 Å distance to the most distal iron-sulfur cluster, called N2, that is located ~15 Å above the membrane surface. The unique Q binding site is a narrow tunnel protruding deep into the enzyme and it is made up of subunits from both arms. The membrane arm extends over 180 Å in the lipid bilayer and provides four putative proton channels. These proton channels are connected to each other and to the Q binding site by a chain of charged amino acid residues located in the center of the membrane arm (Baradaran et al., 2013). Although these findings lay the foundations for a recent and elegant proposal of a proton translocation mechanism by the membrane arm (Kaila, 2018), the process that couples both reactions is still under debate. It was shown experimentally and supported by molecular dynamics (MD) simulations that the redox energy contained in NADH is almost completely transferred to the Q (Sharma et al., 2015; Wikström et al., 2015). This leads to the conclusion that it is the Q chemistry that drives proton translocation by complex I. However, the underlying molecular mechanism remains so far elusive.

More than 20 years ago, when no high resolution structure of complex I was available, the group of Hanns Weiss proposed the presence of a hitherto undetected redox group in the bacterial *Escherichia coli* and the mitochondrial *Neurospora crassa* complex I from UV-vis redox-difference spectroscopy (Friedrich et al., 1998, 2000; Schulte et al., 1998). Complex I is rapidly reduced by NADH preventing the spectral identification of individual redox components (Bakker and Albracht, 1986). To kinetically resolve contributions of the individual redox groups to the UV-vis spectrum, the slower re-oxidation of the reduced enzyme was investigated. These experiments led to the detection of an unknown redox group in complex I called “X” that was reducible with NADH and that was suspected to be involved in electron transfer from cluster N2 to ubiquinone. It was speculated that it might represent a post-translationally modified amino acid (Friedrich et al., 1998, 2000; Schulte et al., 1998). These data fell into oblivion in the course of time. Furthermore, recent structural data showed that such a postulated modified amino acid residue is not present in the complex. However, the important quinone chemistry implies the presence of several quinone species in complex I.

Here, we used a novel protocol for the preparation of *E. coli* complex I to re-investigate the findings obtained more than 20 years ago. The preparation is reduced with NADH and re-oxidized by oxygen from residual atmospheric gas within the assay buffer leading to a kinetic resolution of some of the redox-components. Indeed, just as described, after re-oxidation of the FMN and the Fe/S-clusters, the absorbance of a residual component was detectable. The UV-vis difference spectrum resembles very much the one of the QH^−^ anion. In the presence of the specific Q-site inhibitor piericidin A, the absorbance of this component is significantly diminished. Furthermore, the re-oxidation kinetics of the component is significantly decelerated in the D213G^H^ variant (the superscript denotes the corresponding subunit of *E. coli* complex I). Notably, it is particularly a disturbed Q chemistry that diminishes electron transfer and proton translocation (Nuber et al., manuscript submitted). Both activities showed a significant lag-phase not detected with the wild type complex. This lag-phase is most likely caused by a hampered Q reduction. Unexpectedly, the absorbance of the putative QH^−^ anion was not bleached by the oxidation of neither the wild type nor the variant complex. Based on our data we discuss that a quinol anion is a catalytically important intermediate in the mechanism coupling electron transfer with proton translocation.

## Materials and Methods

### Cell Growth

BW25113Δ*ndh nuo::ntpII FRT* (Burschel et al., 2019) was transformed either with pBAD*nuo*_*his*_*nuoF* containing the *nuo*-operon encoding the wild type enzyme decorated with a His-tag or with pBAD*nuo*_*His*_*nuoF* D213G^H^ encoding the variant. Cells were grown in autoinduction medium while shaking (180 rpm, New Brunswick Innova 44, Eppendorf). At an OD_600_ of ~4.0 cells were harvested by centrifugation (3,913 · g, 15 min, 4°C; JLA 8.1000, Avanti J-26 XP, Beckman Coulter), shock frozen in liquid nitrogen and stored at −80°C.

### Preparation of *E. coli* Complex I

All steps were performed at 4°C. Frozen cells were suspended in A-buffer [1:5 (w/v), 50 mM MES/NaOH, pH 6.0; 50 mM NaCl] supplemented with PMSF (1:1,000, w/v) and 2–3 mg DNase I. Cells were homogenized and disrupted by passing them three times through an EmulsiFlex-C5 (1,000 bar, 4°C, Avestin). The membrane fraction was obtained by differential centrifugation (low speed at 9500 · g, 20 min, 4°C; A8. 24, RC-5 Superspeed Refrigerated Centrifuge, Sorvall Instruments and high speed at 201240 · g, 70 min, 4°C, 60 Ti, L8-M Ultrafuge, Beckman). Membranes were suspended in ${\text{A}}_{6.8}^{*}$-buffer (A-buffer at pH 6.8 plus 5 mM MgCl_2_; 2 mL buffer per g membrane), shock frozen in liquid nitrogen and stored at−80°C. Membrane proteins were solubilized from the membranes by dropwise addition of lauryl maltose neopentyl glycol (LMNG; Anatrace) to a final concentration of 2% (w/v). After 60 min incubation at room temperature, non-solubilized remnants were removed by centrifugation (201,240 · g, 15 min, 4°C, 60 Ti, L8-M Ultrafuge, Beckman). Elution buffer [50 mM MES/NaOH, pH 6.8; 50 mM NaCl; 5 mM MgCl_2_; 0.5 M imidazole; 10% (w/v) glycerol and 0.005% (w/v) LMNG] was added to a final concentration of 20 mM imidazole. Subsequently, the protein was loaded onto a Probond Ni^2+^ affinity chromatography column equilibrated in binding-buffer [50 mM MES/NaOH, pH 6.8; 50 mM NaCl; 5 mM MgCl_2_; 20 mM imidazole; 10% (w/v) glycerol and 0.005% (w/v) LMNG]. After a washing step at 116 mM imidazole, bound proteins were eluted in a single elution step at 308 mM imidazole. Peak fractions were concentrated by ultrafiltration (Amicon Ultra, 100 kDa MWCO; 3,800 · g, 4°C, A-4-44, Eppendorf 5804 R) and loaded onto a Superose 6 size exclusion chromatography column in ${\text{A}}_{\text{MNG}}^{*}$ buffer [A buffer plus 5 mM MgCl_2_; 10% (w/v) glycerol and 0.005% (w/v) LMNG]. Peak fractions of the eluate were concentrated by ultrafiltration, shock frozen in liquid nitrogen and stored at −80°C.

### UV-vis Spectroscopy

UV-vis spectra were recorded with a diode-array UV-vis spectrophotometer (TIDAS II, J&M) using quartz suprasil cuvettes with a path length of 1 cm (Hellma Analytics, Müllheim, Germany). The spectra of the quinone species were recorded in water:ethanol (20:80, v/v) using a 60 μM solution of decyl-ubiquinone (Sigma). Decyl-ubiquinone was reduced at anoxic conditions with a 5-fold molar excess of NaBH_4_ and the solution was titrated to pH 7 with 0.1 M HCl. Decyl-ubiquinol was deprotonated by an addition of 0.2 M NaOH.

For measurements with complex I and the D213G^H^ variant, 1 μM protein in ${\text{A}}_{\text{MNG}}^{*}$-buffer was placed in a stirred cuvette and a spectrum was recorded. The entire sample was set to zero absorbance and a stable baseline was obtained within 30 s. Then, the sample was reduced by an addition of a 10-fold molar excess of NADH (Sigma). Reduction and re-oxidation of complex I was followed by the absorbance at 450 nm and the oxidation of NADH by oxygen dissolved in the buffer at 340 nm. NADH was oxidized faster than the enzyme within about 1 min allowing for the calculation of a redox-difference spectrum of a partly reduced enzyme (Friedrich et al., 2000). To measure the inhibition by piericidin A (Sigma) the protein and the buffer were placed in the stirred cuvette and an ethanolic solution of piericidin A (10 mM) was added. The mixture was incubated at room temperature for 5 min to allow binding of the inhibitor and after that, the reaction was started by an addition of NADH as described above. As control, the equivalent volume of ethanol (10 μL) was added to an aliquot of the preparation and the reaction was started after 5 min incubation.

### Analytical Procedures

The NADH/ferricyanide oxidoreductase activity was measured as decrease of the ferricyanide concentration over time at 410 nm (macro cuvette QS, d = 1 cm, Hellma Analytics; TIDAS II, J&M Analytik AG). One milliliter ferricyanide (1 mM final concentration) in A^*^-buffer was mixed with 200 μM NADH and after the onset of a stable baseline, the reaction was started by adding 5 μL of the fractions of the chromatographic columns. The NADH/ferricyanide oxidoreductase activity was calculated according to the Lambert–Beer law using a molar extinction coefficient for ferricyanide of 1 mM^−1^. cm^−1^ (Friedrich et al., 1989). SDS polyacrylamide gel electrophoresis (SDS-PAGE) was performed according to Schägger and von Jagow (1987). NADH:decyl-Q oxidoreductase activity and proton translocation measured as quench of the 9-amino-6-chloro-2-methoxyacridine (ACMA) fluorescence were determined as described (Mühlbauer et al., 2020). The proton gradient across the proteoliposome membrane was dissipated by an addition of 10 μM carbonyl cyanide 3-chlorophenylhydrazone (CCCP).

## Results

### Spectrum of a Redox Component in *E. coli* Complex I Beyond Cluster N2

**Figure 1:**
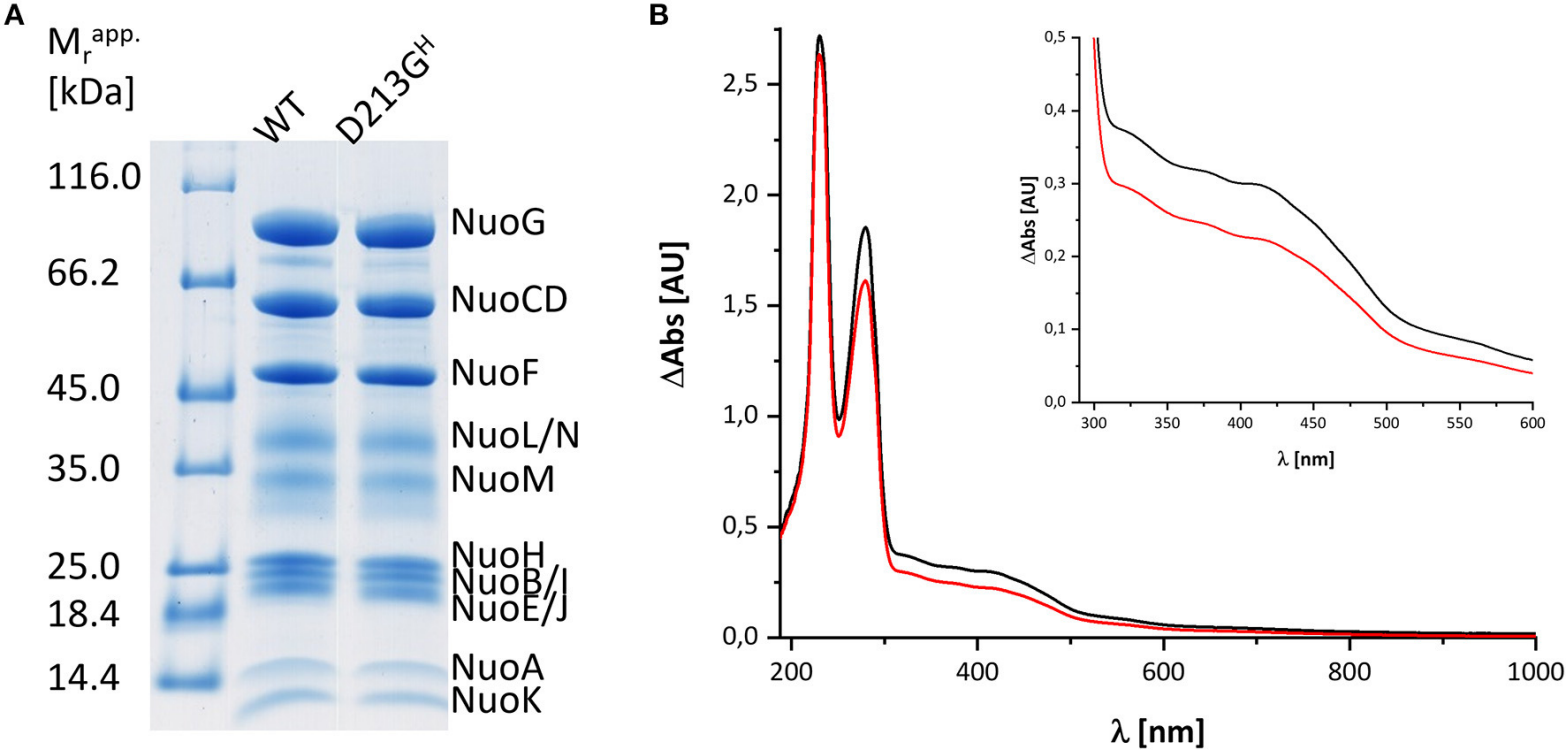
Characterization of the preparations used in this study. **(A)** SDS-PAGE of the preparations of wild type (WT) and the D213G^H^ variant. The bands are attributed to individual subunits according to their apparent molecular mass. The left lane shows the bands of the marker with the individual masses in kDa. **(B)** UV-vis spectra of the preparations of the wild type (black) and the D213G^H^ variant (red). The inset shows the spectral region with the absorbance of the cofactors with a magnified scale.

Bacterial complex I from *E. coli* is made up of 13 different subunits that are encoded in the *nuo*-operon. In *E. coli*, the genes *nuoC* and *nuoD* are fused to a single gene *nuoCD* (Braun et al., 1998). We routinely use a host strain that chromosomally lacks the *nuo*-genes and that is transformed with a pBAD overexpression plasmid containing the entire *nuo*-operon, facilitating manipulation of the *nuo*-genes (Mühlbauer et al., 2020). For fast enzyme purification, the sequence of a His-tag is fused N-terminally to NuoF. A stable and homogeneous preparation of the complex produced from the *nuo*-operon on the plasmid is obtained in the presence of the detergent LMNG by affinity- and size-exclusion-chromatography resulting in sufficient material of excellent quality for spectroscopic analysis (Figure 1). The preparation differs in nearly all aspects from that used for the spectroscopic investigations 20 years ago: First, both the host strain and the expression plasmid used to produce the complex are different and second, chromatography media and the detergent have changed. To address the question whether the UV-vis redox-difference spectrum of the novel preparation of the *E. coli* complex I still provides an indication for a component that is neither the FMN nor an Fe/S cluster, we repeated the experiments following the re-oxidation of a preparation that was reduced with NADH.

**Figure 2:**
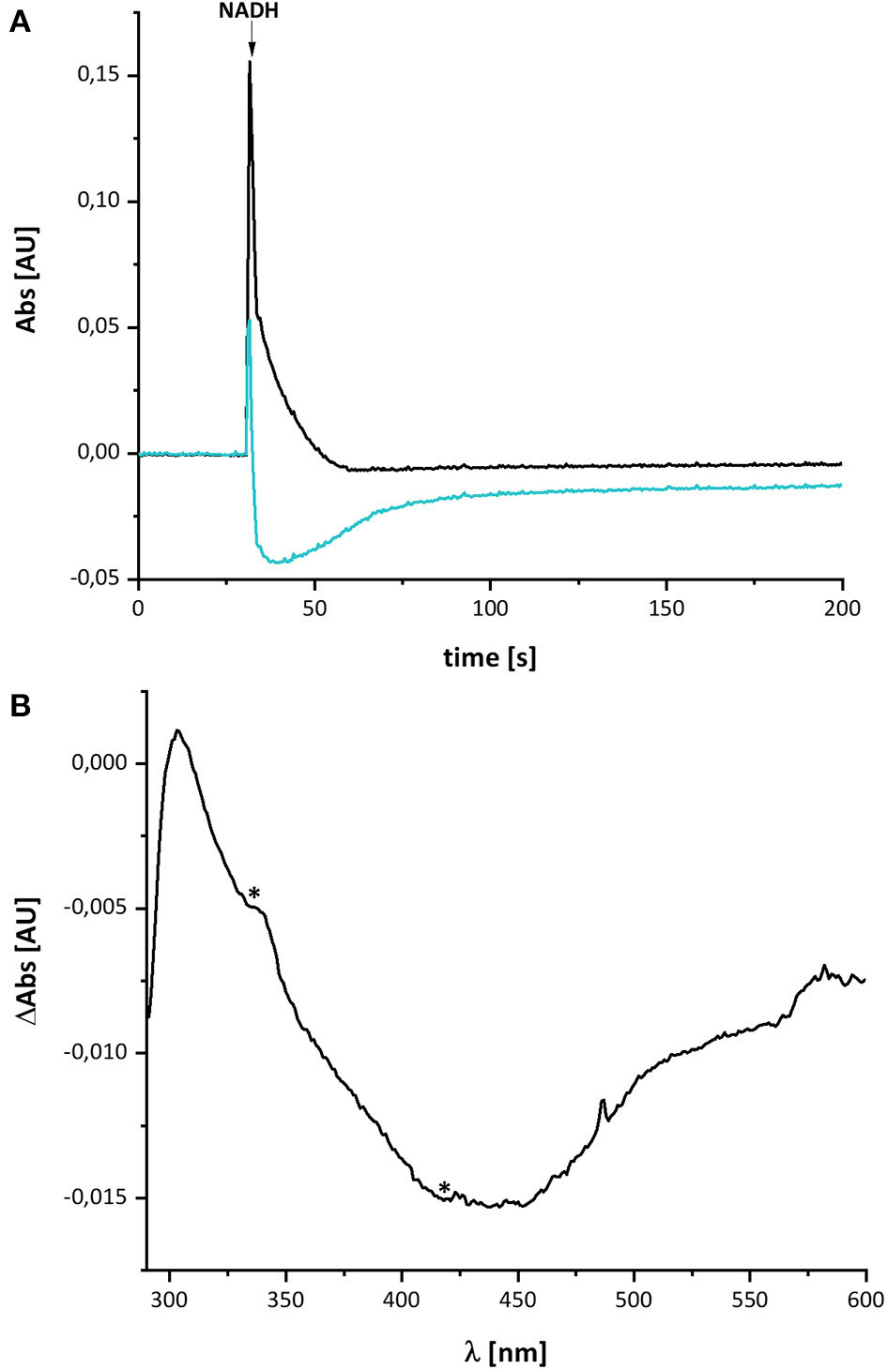
Reduction of *E. coli* complex I by NADH followed by partial re-oxidation by oxygen from the air. **(A)** The black trace shows the absorbance of NADH at 340 nm and the blue trace that of the complex I at 450 nm. **(B)** Shows the (reduced-minus-oxidized) difference spectrum observed after 2 min after the addition of NADH. Residual contributions from the two tetranuclear Fe/S clusters on NuoI at 334 and 418 nm are indicated by asterisks.

Figure 2 shows the reduction of complex I by NADH and its subsequent re-oxidation by oxygen from the air dissolved in the buffer. The oxidation of NADH is followed at 340 nm and the redox state of the complex by its absorbance at 450 nm. Although, the absorption of the redox groups of complex I is partly masked by the spectral contributions from NADH at the beginning of the reaction, this changes after ~1 min, when the added NADH is fully oxidized: while the spectrum lacks potential contributions from NADH at 340 nm being indicative for full NADH oxidation, residual features between 290 and 600 nm suggest that the complex is still partly reduced (Figure 2B). The additional absorbance with two broad negative peaks at 334 and 418 nm that extends up to 550 nm derives from the two tetranuclear Fe/S clusters on NuoI (Rasmussen et al., 2001). Admittedly, the strong positive absorbance of the remaining NAD^+^ dominates the spectrum at wavelength shorter than 290 nm. However, the spectral range from 290 to 600 nm remained unaffected by NAD^+^ accumulation rendering the redox-difference spectrum in that range suitable for a reliable evaluation (Figure 2). The spectrum is characterized by a positive peak around 305 nm and a broad negative absorbance around 440 nm. Thus, the UV-vis difference spectrum obtained with the new preparation looks very similar to that detected in *N. crassa* and *E. coli* complex I more than 20 years ago. Noteworthy, the absorbance at 450 nm does not return to its original zero value, opposed to what was reported in the early experiments. This might be due to the capability of the novel preparation to catalyze the energy conversion step (Mühlbauer et al., 2020). The pmf that is lacking in this experiment would therefore be needed to drive the reverse reaction. The capability of the old preparations to catalyze proton-coupled electron transfer was never measured. We will further elaborate on this below.

### Spectra of Various decyl-Ubiquinone Species

**Figure 3:**
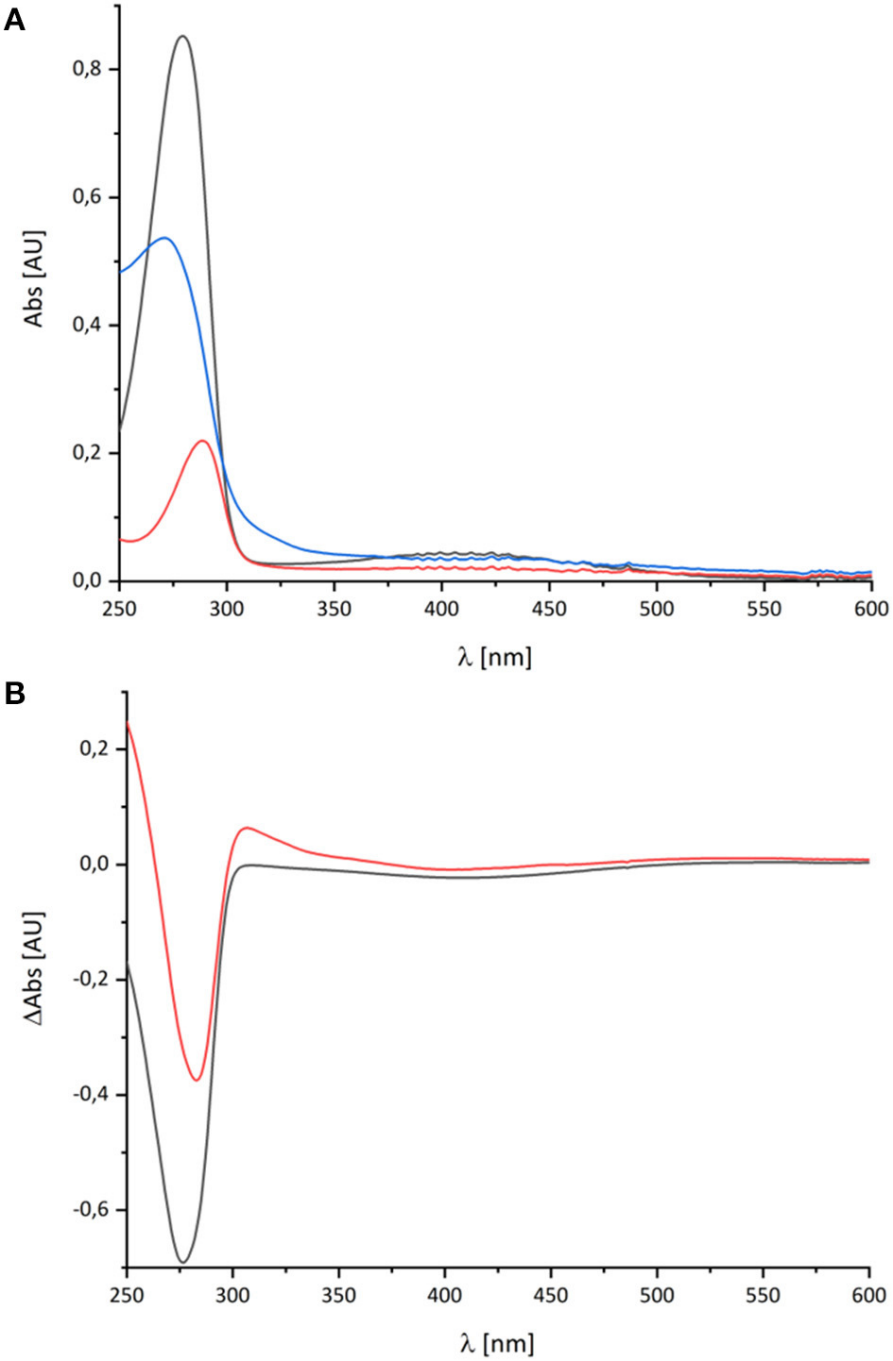
UV-vis spectra of decyl-Q. **(A)** Absorbance spectra of 60 μM decyl-Q in water:ethanol (20:80, v:v; black), after addition of NaBH_4_ and adjustment of pH to 7.0 resulting in the formation of decyl-QH_2_ (red) and after addition of 0.2 M NaOH (pH ~ 13) resulting in the formation of decyl-QH^−^ anion (blue). **(B)** Shows the (reduced-minus-oxidized) difference spectrum of (quinol-minus-quinone) in black and (quinol anion-minus-quinone) in red.

The spectral signature of a component that stayed partly reduced after re-oxidation of NADH-reduced complex I indicates the presence of another component in complex I beyond the FMN and the Fe/S-clusters. In the original publications, it was proposed that this component may reflect the presence of a redox-active post-translationally modified amino acid such as a quinoid group found in quinoproteins (Friedrich et al., 1998, 2000; Schulte et al., 1998; Williamson et al., 2014). Recently, the structure of ovine complex I was determined at 2.3 Å resolution (Kampjut and Sazanov, 2020). At this resolution, the structure actually shows that complex I does not contain a posttranslationally modified amino acid residue with a quinoid structure. However, the simplest explanation for the UV-vis spectrum is that it represents a Q species tightly bound to complex I. This seems feasible as our preparation contains about 0.5 mol Q/mol enzyme. Furthermore, the spectrum of the unknown component is reminicent of the spectrum of the ubiquinol anion that has been recorded in water/ethanol (20:80, v:v; Rich and Bendall, 1980). To compare the spectra of the quinone species with the spectrum of the unknown redox component detected in complex I, we recorded the UV-vis spectra of decyl-Q in (20:80, v:v) water/ethanol with the same diode array photometer (Figure 3).

As reported in the literature, the oxidized Q featured an absorbance maximum at 280 nm and a broad positive absorbance around 420 nm (Figure 3). This very sample was reduced by an addition of NaBH_4_ and titrated to pH 7.0 by 0.1 M HCl leading to the typical QH_2_ spectrum with a maximum at 290 nm and a very minor broad positive absorbance from 360 to 480 nm (Figure 3). Deprotonating this species by adding 0.2 M NaOH resulted in a pH of about 13 and, consecutively, to the formation of the QH^−^ anion. The UV-vis spectrum of the QH^−^ anion is dominated by a maximum at 275 nm and a broad positive absorbance in the vis-region (Figure 3).

**Figure 4:**
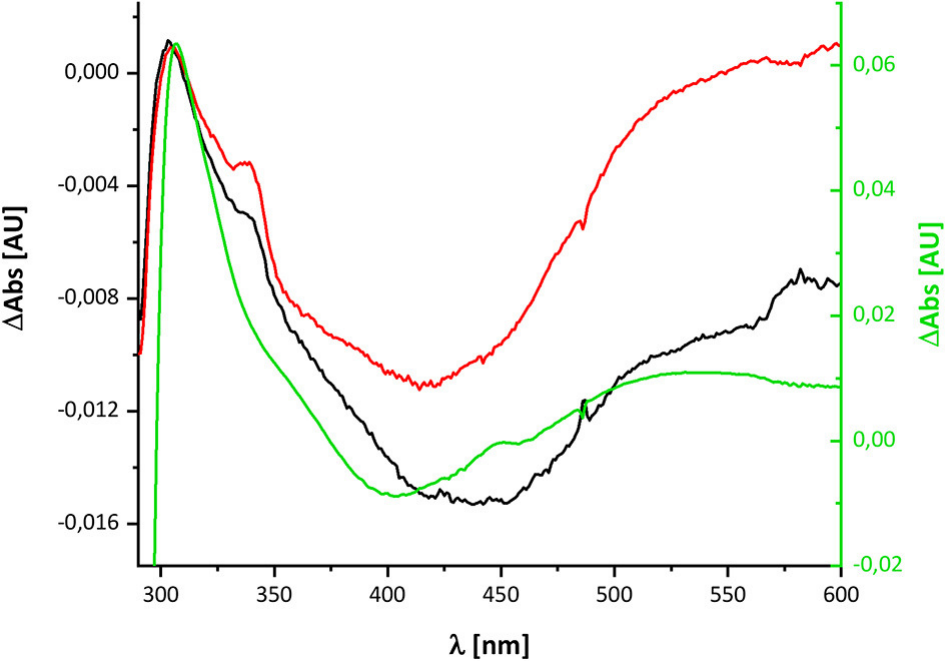
Comparison of the redox-difference spectra of the component detectable after NADH re-oxidation in wild type in black and D213G^H^ variant in red and that of the quinol anion in green.

The UV-vis spectrum of the component detected in complex I represents its (reduced-minus-oxidized) difference spectrum (Figure 2). Therefore, we calculated the (quinol-minus-quinone) and the (quinol anion-minus-quinone) difference spectra (Figure 3). It turned out that the difference spectrum detected in complex I is rather similar to the calculated difference spectrum of the QH^−^ anion minus the quinone. It shows a positive peak at 307 nm and a broad negative absorbance between 330 and 600 nm (Figure 3). Thus, it is reasonable to assume that the catalytic intermediate detected in the reaction cycle of complex I is a QH^−^ anion (Figure 4).

### Re-oxidation of the NADH-Reduced D213G^H^ Variant

**Figure 5:**
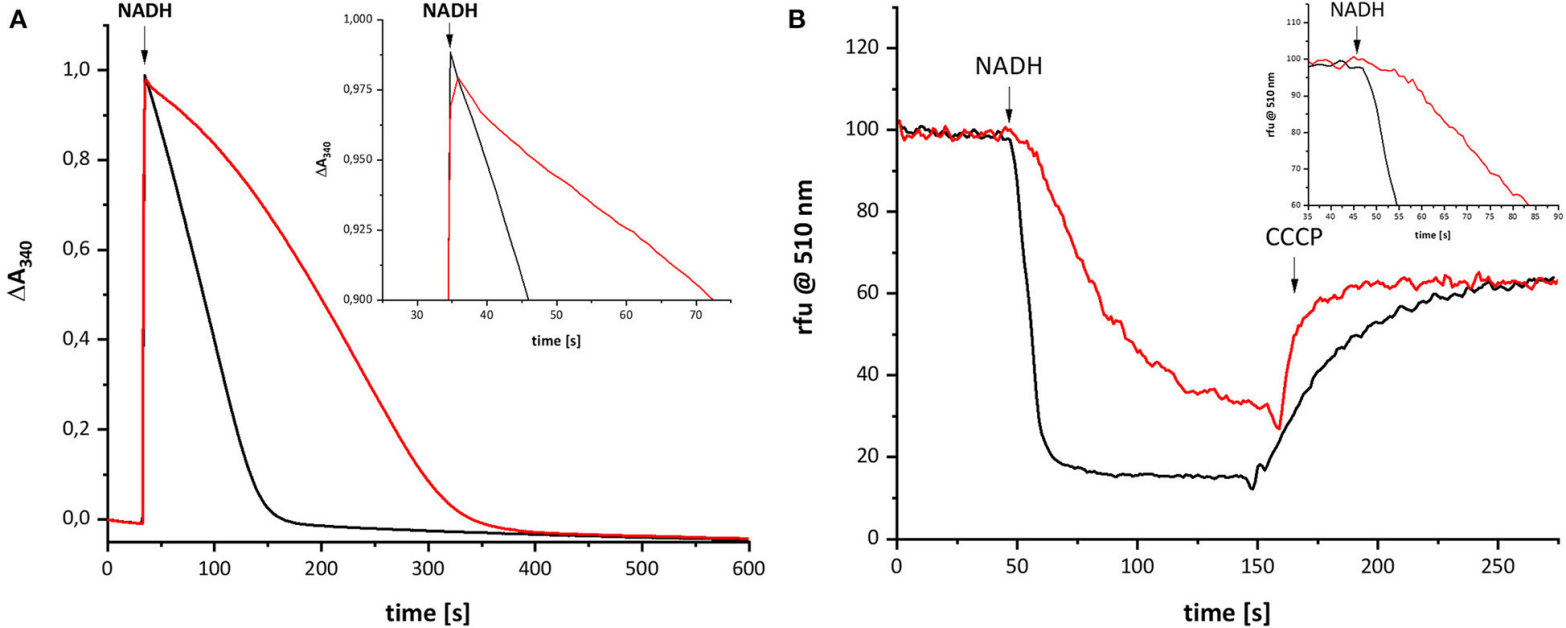
Catalytic activities of wild type complex I (black) and the D213G^H^ variant (red). **(A)** Shows the NADH:decyl-Q oxidoreductase activity and **(B)** the quench of the ACMA fluorescence after reconstitution of the preparations into proteoliposomes. The proton gradient established by the catalytic activity of complex I is dissipated by an addition of carbonyl cyanide 3-chlorophenylhydrazone (CCCP). The insets show the initial activities with a magnified scale.

Recently, we characterized a complex I variant that showed a prominent lag-phase in activity attributed to a disturbed Q reduction (Nuber et al., manuscript submitted). In humans, the mutation D199G within the mitochondrially encoded subunit ND1 was detected in a patient, who suffered from exercise intolerance and nephropathy in his 40 s. This position is homologous to position D213 on subunit NuoH in *E. coli* complex I (Supplementary Figure 1). The corresponding mutation was introduced into *E. coli*, resulting in the D213G^H^ variant. The variant was stably assembled and enzymatically active although with a diminished reaction rate. Most importantly, the D213G^H^ variant showed a prominent lag-phase in electron transfer and proton translocation and its maximal turnover was reached only after ~1 min (Figure 5). By contrast, the wild type enzyme is active in both respects right after substrate addition. It might be possible that the disturbed Q chemistry of the variant is related to the reaction of the putative QH^−^ anion and, hence, that its re-oxidation is also delayed to the same extent as the electron transfer and proton translocation activity.

**Figure 6:**
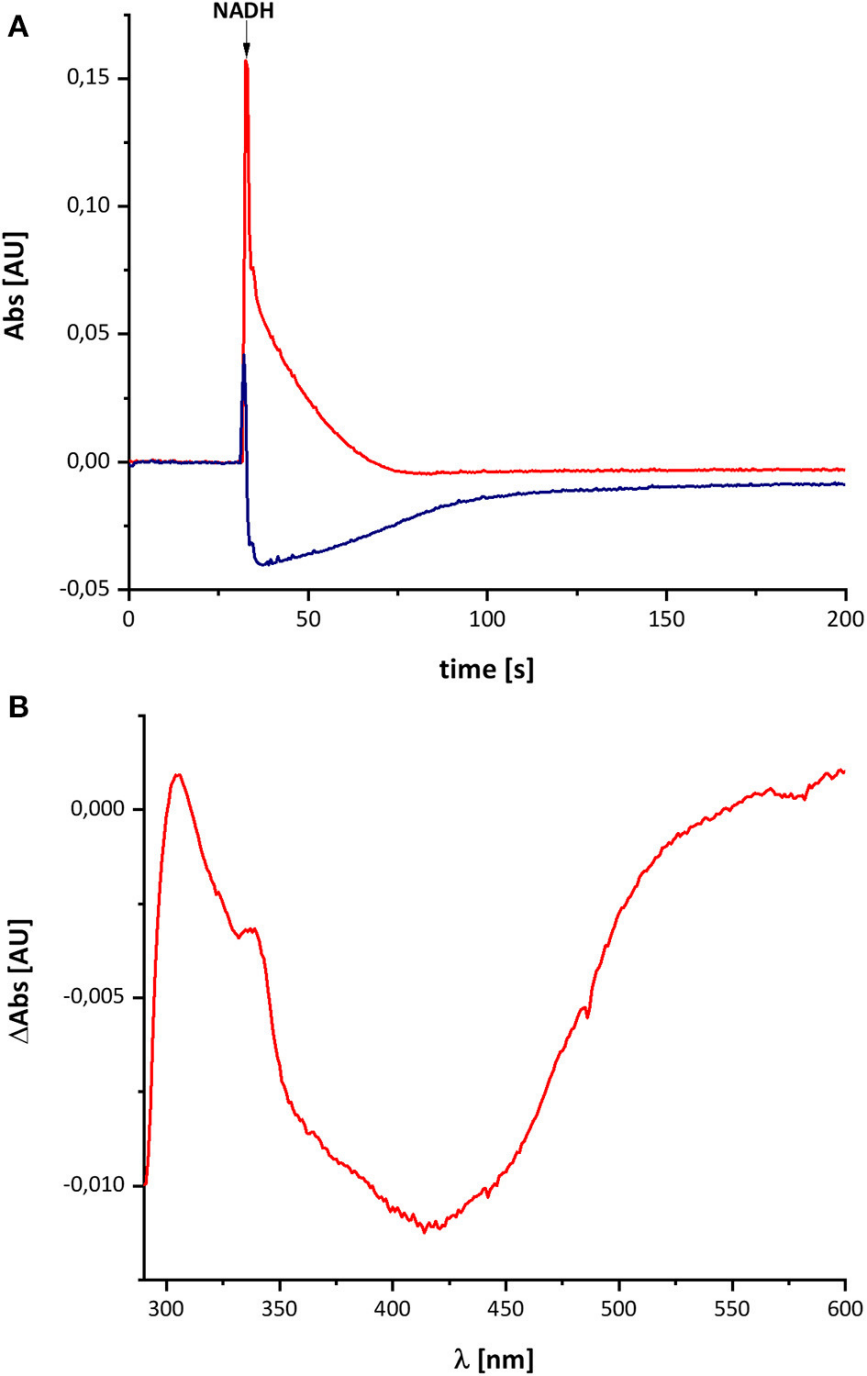
Reduction of the D213G^H^ variant by NADH followed by partial re-oxidation by oxygen from the air. **(A)** The red trace shows the absorbance of NADH at 340 nm and the blue trace that of the complex I at 450 nm. **(B)** Shows the (reduced-minus-oxidized) difference spectrum observed after 2 min after the addition of NADH.

**Figure 7:**
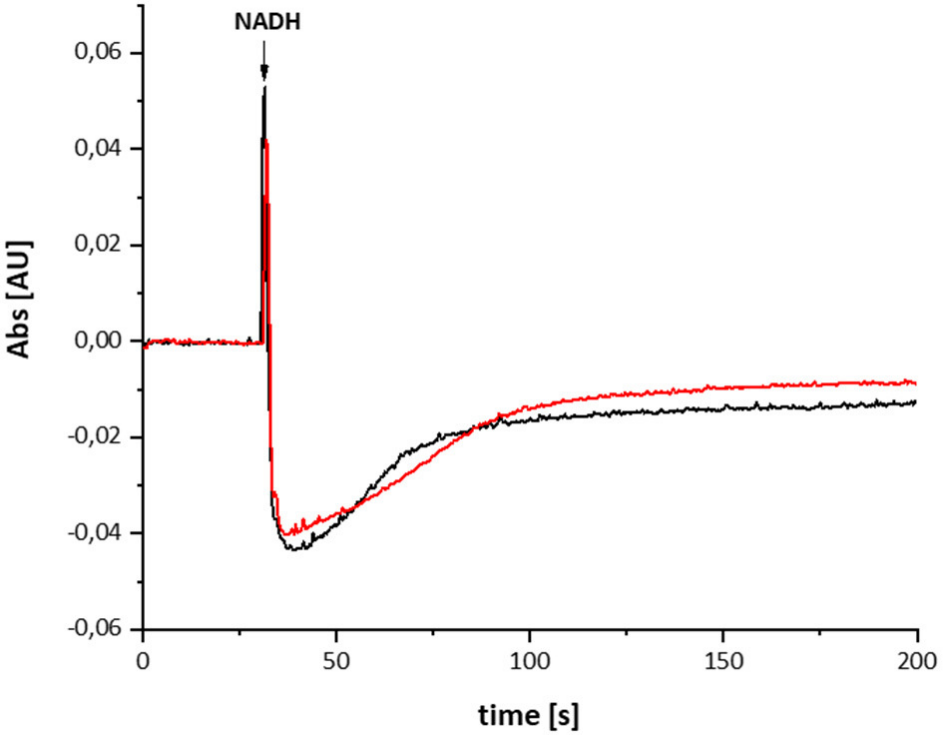
Reduction of the wild type complex (black) and the D213G^H^ variant (red) by NADH. The trace of the absorbance at 450 nm is shown. The variant re-oxidizes significantly slower than the wild type enzyme.

The variant was prepared from the mutant strain in full analogy to the procedure applied to the parental strain. Not surprisingly, the SDS-PAGE and UV-vis spectrum of the preparation were virtually identical to that of the wild type complex (Figure 1). This sample was subjected to the same procedure of reduction and re-oxidation as the wild type complex (Figure 6). The UV-vis difference spectrum obtained with the variant also featured the positive peak around 305 nm and the broad negative absorbance around 440 nm. Thus, the difference spectra obtained with the wild type complex and the D213G^H^ variant are very similar to each other (Figures 4, 6). As observed for the wild type, the variant was quickly reduced by NADH and rapidly re-oxidized within 60 sec (Figure 6). Just as before, the absorbance at 450 nm did not return to its original value suggesting an incomplete re-oxidation of the NADH-reduced variant. While both preparations, wild type and variant, are reduced to nearly the same extend, a small difference in the minimum of the two traces recorded at 450 nm after NADH addition was observed, presumably caused by slight differences in enzyme concentration (Figure 7). Remarkably, however, the variant is re-oxidized much slower than the original enzyme (Figure 7). Thus, not only the forward, but also the reverse reaction is hampered in the variant.

### Effect of Piericidin A on the Redox-Difference Spectrum

**Figure 8:**
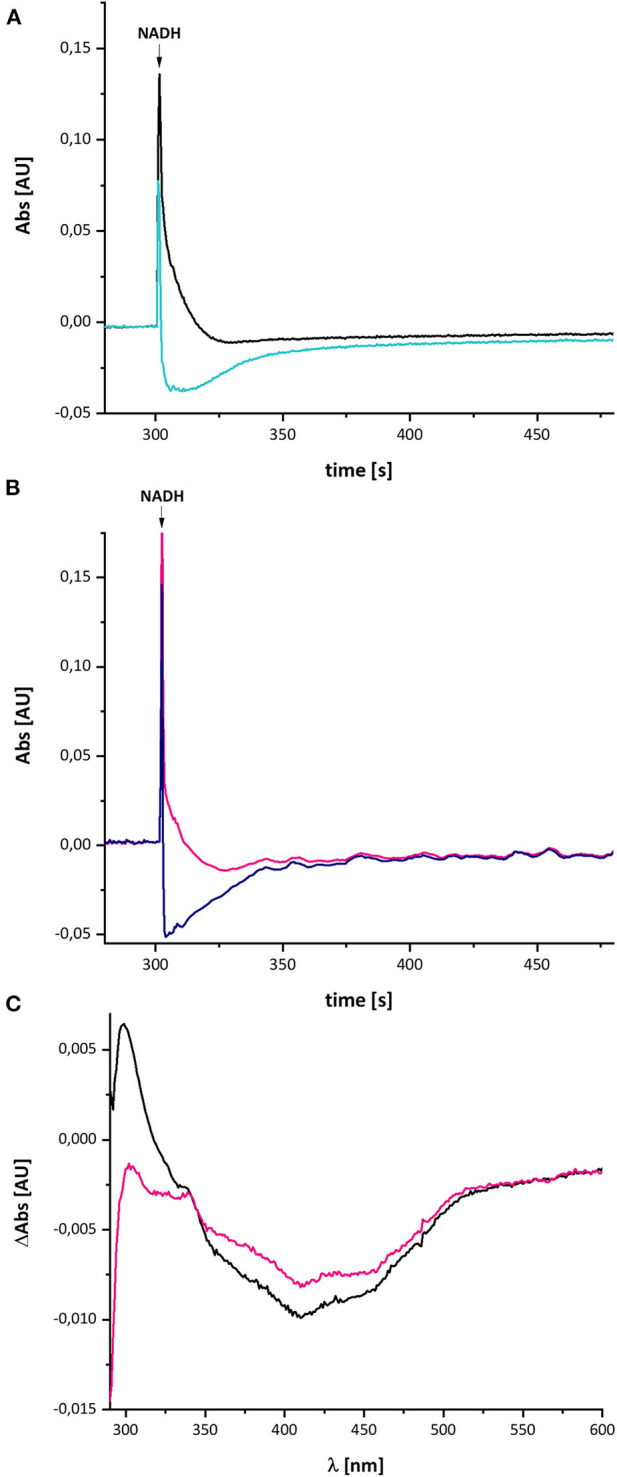
Reduction of *E. coli* complex I, treated either with ethanol **(A)** or with piericidin A **(B)**, by NADH followed by partial re-oxidation by oxygen from the air. **(A)** The black trace shows the absorbance of NADH at 340 nm and the light blue trace that of the ethanol-treated complex I at 450 nm. **(B)** The pink trace shows the absorbance of NADH at 340 nm and the dark blue trace that of the piericidin A-treated complex I at 450 nm. The curves look a bit disturbed due to some dust in the cuvette. **(C)** Shows the (reduced-minus-oxidized) difference spectrum of the ethanol-treated (black) and the piericidin A-treated (pink) complex observed after 2 min after the addition of NADH.

In case the UV-vis difference spectrum indeed reflected the formation of a quinol anion within the Q binding-cavity, the spectrum should be influenced by an addition of a specific Q-site inhibitor such as piericidin A (Bridges et al., 2020). We performed the assay as described above with complex I in a buffer containing 10 μM piericidin A and used an aliquot of the same preparation incubated with the equivalent volume of ethanol as control (Figure 8). Due to the lack of lipids and a Q regenerating system in the assay, the *E. coli* complex I exhibits a rather low activity of 2.5 U/mg protein. This activity is inhibited to 60% (1.57 U/mg protein) by an addition of 10 μM piericidin A under these assay conditions. The piericidin A treated complex was quickly reduced to the same extent as the ethanol-treated control but it re-oxidized substantially faster. The UV-vis difference spectrum of the inhibited complex is similar to the one obtained from the ethanol-treated control, but its peaks are significantly smaller, although both measurements were performed using the same protein concentrations. Relative to the absorbance at 600 nm, the positive peak at 305 nm was about one fifth of that of the control (Figure 8). The broad negative absorbance from 350 to 550 nm is diminished only by about 20%. This is due to the strong residual absorbance of the two Fe/S clusters on NuoI that mainly contribute to this spectral region (Rasmussen et al., 2001). The absorbance at 305 nm is not completely bleached by 10 μM piericidin A due to the incomplete inhibition.

In contrast to the other measurements, the absorbance at 450 nm of the piericidin A treated enzyme returned to its original value, indicating that the NADH-reduced complex is fully re-oxidized. A possible explanation for this finding is provided in the section Discussion.

## Discussion

Here, we have re-investigated the UV-vis spectroscopic experiments from the Hanns Weiss lab that were conducted more than 20 years ago (Friedrich et al., 1998, 2000; Schulte et al., 1998). We were able to reproduce the data with a completely novel preparation of the *E. coli* complex I and obtained a UV-vis redox-difference spectrum of a complex I component highly similar to the one that was called “X” in the first reports (Figures 2, 5, 6). The spectrum is characterized by a positive absorption band around 305 nm and a very broad negative absorbance around 425 nm (Figures 4, 8). In the past, the spectrum was interpreted to derive from a modified amino acid that mediates electron transfer between the distal Fe/S-cluster N2 and the substrate Q. However, recent insights into the molecular structure of complex I from various organisms by X-ray crystallography and cryo-EM render this assumption obsolete (Agip et al., 2019). A simple answer to the question of the molecular identity of the group “X” is that it represents a quinol anion that is playing a role in the catalytic cycle of complex I. The postulate of a QH^−^ anion is supported by the similar UV-vis redox-difference spectra of the group “X” and of the QH^−^ anion (Figures 3, 4; Rich and Bendall, 1980). In addition, two semiquinone radicals were reported to be involved in the reaction cycle of complex I (Ohnishi et al., 2018). However, the signal of the dominant species called Q_Nf_ depends on the presence of the pmf. Here, the enzyme was investigated in detergent, not in proteoliposomes, spheroplasts or SMPs, hence in the absence of a pmf. Consequently, radical signals were not observed (De Vries et al., 2015). The quinone species detected here can, thus, not reflect a radical state of the quinone.

**Figure 9:**
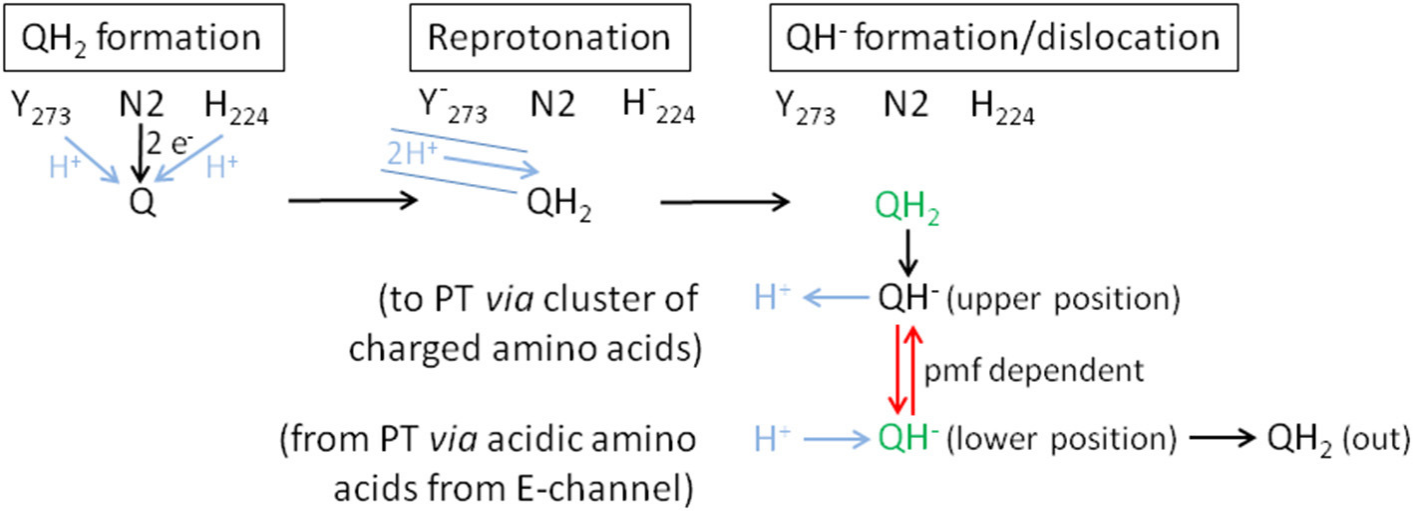
Proposed mechanism of coupling electron transfer with proton translocation in complex I. Proton transfer reactions are shown in blue, the pmf-dependent dislocation of the quinol anion in the Q cavity in red and the positions that are blocked by piericidin A in green. Q is fully reduced close to Fe/S cluster N2 and protonated by Tyr273^CD^ and His224^CD^ (QH_2_ formation). This position (green) is partly blocked by piericidin A. The recently discovered proton pathway in *Yarrowia lipolytica* complex I (Grba and Hirst, 2020) is indicated by the blue solid lines (Reprotonation). The 2 protons indicated in the pathway are either used to re-protonate the reactive amino acids or the quinone directly. Formation of QH_2_ leads to its minor movement to the cluster of charged amino acids (Sharma et al., 2015) caused by breaking the hydrogen bond between H224^CD^ and D325^CD^ (QH^−^ formation/dislocation). At the cluster of charged amino acids (upper position) QH_2_ is deprotonated to the QH^−^ anion and the proton enters the membrane to initiate proton translocation (PT) according to the proposed mechanism (Kaila, 2018). To finish proton translocation the surplus proton is taken up by the QH^−^ anion that is displaced to the lower position. This position (green) is also blocked by piericidin A. The displacement of the QH^−^ anion is the energy releasing step. In the reverse reaction, the QH^−^ anion needs the pmf to be placed at the upper position. In the absence of the pmf, as in our experiments, the QH^−^ anion stays at the lower position and is therefore detectable by UV-vis spectroscopy.

In addition, it was published that signals of the Q methoxy groups are expected to be detectable in the FT-IR difference spectrum when reducing the oxidized Q to QH_2_ (Hellwig et al., 1999). Initially, we speculated that the unknown group acts as a converter of two one-electron-transfers to one two-electron transfer reaction, implying its transition from the oxidized to its fully reduced form. Due to the lack of IR modes specific for the methoxy groups in previously published FT-IR redox difference spectra (Friedrich et al., 2000), we concluded at that time that the unknown redox group is not Q bound to complex I. Here, we now propose that the UV-vis difference spectrum reflects the generation of a quinol anion (Figure 9). Importantly, this non-redox process will not cause the raise of the signals of the methoxy groups (Hellwig et al., 1999), so that the published FT-IR difference spectrum of the unknown group (Friedrich et al., 2000) is in line with the proposal that the spectrum stems from a quinol anion. The interpretation of the UV-vis difference spectrum as QH^−^ anion is further supported by the significant decrease of its spectral amplitude in the presence of the Q-site specific inhibitor piericidin A (Figure 8). As piericidin A competes with Q for the same binding site (Bridges et al., 2020; Gutiérrez-Fernández et al., 2020), a smaller signal is expected.

The difference between the re-oxidation kinetics reported in the previous work from the Weiss group and those presented here is the incomplete re-oxidation of the NADH-reduced complex over time. In the old experiments, after reduction of the enzyme by either NADH or dithionite, the complex was fully oxidized by atmospheric oxygen. In the experiments shown here, the residual absorbance of the putative QH^−^ anion is detectable for more than 10 min and does not disappear (Figures 2, 6). We hypothesize that the perseverance of the absorbance is connected to the capability of the new preparation to catalyze the energy converting step of the overall reaction (Mühlbauer et al., 2020), a property that has not been investigated with the preparations 20 years ago. Indeed, we propose that the QH^−^ anion is a catalytic intermediate of the reaction cycle coupling electron transfer and proton translocation in the complex. We suggest that coupling is mediated by the QH^−^ anion, which has to change its position in the quinone binding cavity to become protonated in the reverse reaction and then re-oxidized as will be explained below. Since this change in position would be the energy converting step, its reversal depends on the presence of the pmf. Thus, the QH^−^ anion is not re-oxidized in our experiments due to lack of a driving force (Figure 9).

According to MD simulations the first and second electron transfer reactions from Fe/S-cluster N2 to Q are isoenergetic leading to a redox potential of bound ubiquinone of about −260 mV (Sharma et al., 2015; Gamiz-Hernandez et al., 2017; Warnau et al., 2018). This is close to the experimentally determined value of about −295 mV (Hielscher et al., 2013) and < -300 mV (Verkhovskaya and Wikström, 2014). Consequently, it is generally accepted that proton translocation is driven by the quinone chemistry. Structural data and MD simulations imply that the quinone binding cavity comprises at least two positions for quinone binding (Verkhovsky et al., 2012; Baradaran et al., 2013; Wikström et al., 2015; Haapanen et al., 2019; Parey et al., 2019; Grba and Hirst, 2020; Kampjut and Sazanov, 2020). The substrate Q binds in 12 Å distance to cluster N2. Its quinoid head group is hydrogen-bonded by the conserved residues Y273^CD^ and H224^CD^ (Tocilescu et al., 2010). In this position, Q is reduced by sequential electron transfer from cluster N2 and protonated by these two residues. We propose that the Q is fully reduced and protonated to QH_2_ at this site (Figure 9). MD simulations suggest that formation of the anionic form of the two amino acid residues binding Q induces conformational changes enabling a path to the E-channel (Gutiérrez-Fernández et al., 2020). A recently discovered water-channel may be used to re-protonate the involved tyrosine and histidine residues (Grba and Hirst, 2020).

Protonation of Q by H224^CD^ leads to the breaking of a hydrogen bond between H224^CD^ and the neighboring D325^CD^ (Sharma et al., 2015; Warnau et al., 2018; Kampjut and Sazanov, 2020). This induces a conformational move enabling the dissociation of QH_2_ that then most likely moves toward a nearby cluster of charged amino acids. It was proposed that QH_2_ movement may initiate proton pumping (Sharma et al., 2015) by a change of dipolar interactions within the membrane arm (Kaila, 2018). The cluster of charged amino acid residues connects the quinone binding site with the E-channel, the putative proton translocation pathway closest to the peripheral arm. MD simulations and cryo-EM show that this region is hydrated (Haapanen and Sharma, 2017; Grba and Hirst, 2020). Electrostatic calculations indicate that the movement of QH_2_ may change the protonation state of nearby titratable residues (Sharma et al., 2015; Gamiz-Hernandez et al., 2017; Haapanen and Sharma, 2017; Warnau et al., 2018; Haapanen et al., 2019), possibly enabling the deprotonation of QH_2_ to the QH^−^ anion that we propose to be a relevant intermediate. The cluster of charged amino acids might thus function as a proton relay system that deprotonates QH_2_. Position D213^H^ is part of the cluster of charged amino acid residues, providing a possible explanation of the disturbed Q reduction in the D213G^H^ variant (Nuber et al., manuscript submitted). The proton released from QH_2_ in its “upper position” is subsequently transferred to the E-channel leading to a “proton push” that initiates a cascade of changing dipolar interactions, which is transmitted all along the membrane arm (Kaila, 2018). The “wave” of changing dipolar interactions is reflected at the end of the membrane arm and travels back toward the peripheral arm, leading to proton uptake from the N-side and to proton release to the P-side (Kaila, 2018). This process ends with a surplus proton that might be localized on an acidic amino acid residue of the E-channel. According to our proposal, the QH^−^ anion formed at the cluster of charged amino acids moves to its second Q binding site, the “lower position,” in the quinone binding cavity close to the E-channel leading to a “proton pull.” Here, the surplus proton from one of the acidic amino acids it taken up by the QH^−^ anion and the resulting QH_2_ finally leaves the quinone binding cavity.

Remarkably, the absorbance at 450 nm of complex I returns to its original value about 3 min after NADH addition only in the presence of piericidin A (Figure 8). We interpret this finding as the capability of piericidin A to also block the second QH^−^ anion binding site (its “lower position”), preventing its displacement within the Q cavity. A second binding site for piericidin A was modeled in mouse complex I (Bridges et al., 2020). This binding site roughly corresponds to the position of the second Q binding site in *Thermus thermophilus* (Warnau et al., 2018) and *Yarrowia lipolytica* (Haapanen et al., 2019; Parey et al., 2019). According to our proposal, the QH^−^ anion is re-protonated at this “lower position.” We propose that in the presence of piericidin A, a residual amount of QH^−^ is formed in the “upper position,” however, the anion cannot move to the “lower position” that is blocked by the inhibitor. Correspondingly, the QH^−^ anion can be fully re-oxidized in the reverse reaction at the “upper position” (Figure 9). In the reverse reaction, it will gain a proton from the cluster of charged amino acids and can be de-protonated and re-oxidized in the position close to cluster N2 without the need of the pmf enabling its shift to the “upper position” (Figure 9).

According to our hypothesis, the movement of the QH^−^ anion is the energy converting step in complex I. This is a variation of the two-stroke model proposed by Brandt (2011) in which the semiquinone anion (Q^•−^) is transferred by conformational changes from a destabilizing to a stabilizing state inducing the translocation of two protons. In the next step the QH^−^ anion is formed in an environment leading to a pKa of the QH^−^ anion of about 11.5. The transfer to a different environment by a conformational change results in a pKa of about 8. The second transition is connected to the translocation of another two protons (Brandt, 2011). This “two-stroke” mechanism requires the presence of only two proton pathways in the membrane arm. Accordingly, our model is a “one stroke” model in which the energy obtained from the displacement of the QH^−^ anion drives translocation of four protons through four proton pathways (one proton per pathway) according to the mechanism provided by Kaila (2018). The latter mechanism, however, needs to be initiated by a yet unknown “proton push” and depends on a “proton pull” at the end of one catalytic cycle. Here, we propose two different states of the QH^−^ anion at two different binding positions and assume that its movement provides the energy for proton translocation. The “proton push” initiated by the formation of the QH^−^ anion places the electrostatic interactions of the charged amino acids in the membrane arm in a position, which allows for a loaded spring type mechanism, so that the “proton pull” resulting in the protonation of the QH^−^ anion ultimately leads to proton translocation. In order to achieve this, the QH^−^ anion has to change its position in the quinone binding cavity from the position close to cluster N2 (“upper position”) to a position close to the E-channel (“lower position”). In terms of the temporal arrangement of these steps within the reaction cycle of complex I, the initial reduction of the complex by NADH leads to the formation of QH_2_ at the upper position. Now, the quinol is deprotonated at the cluster of charged amino acids including position D213 and the QH^−^ anion moves to the lower position. If electrons are now withdrawn from the complex at the NADH binding site, for instance through the action of molecular oxygen, the FMN and the Fe/S clusters are re-oxidized. However, this has no impact on the QH^−^ anion as it cannot move to the upper position due to the lack of the pmf. That explains why the UV-vis absorbance of the QH^−^ anion remains after NADH, FMN and the Fe/S clusters have been oxidized. As a consequence of our model, energy coupling in complex I does not involve a previously proposed electron transfer step (Brandt, 2011; Wikström et al., 2015), but instead is solely mediated by a sequence of de-protonation and protonation reactions and the movement of the quinol anion.

## Data Availability Statement

The raw data supporting the conclusions of this article will be made available by the authors, without undue reservation.

## Author Contributions

FN and TF recorded and calculated the enzyme spectra. LM and TF recorded and calculated the quinone spectra. JS established the novel preparation protocol. FN purified the enzymes. FN and SO made the variant. TF and DW wrote the manuscript with the help of all authors and TF designed the study. All authors contributed to the article and approved the submitted version.

## Dedication

Dedicated to Prof. Dr. Hanns Weiss on the occasion of his 80^th^ birthday.

## Conflict of Interest

The authors declare that the research was conducted in the absence of any commercial or financial relationships that could be construed as a potential conflict of interest.
