# Supplementary figures and images for: A Quinol Anion as Catalytic Intermediate Coupling Proton Translocation With Electron Transfer in E. coli Respiratory Complex I
Source: Front Chem. 2021 May 7;9:672969. doi: 10.3389/fchem.2021.672969 (PMC8138167; doi:10.3389/fchem.2021.672969)

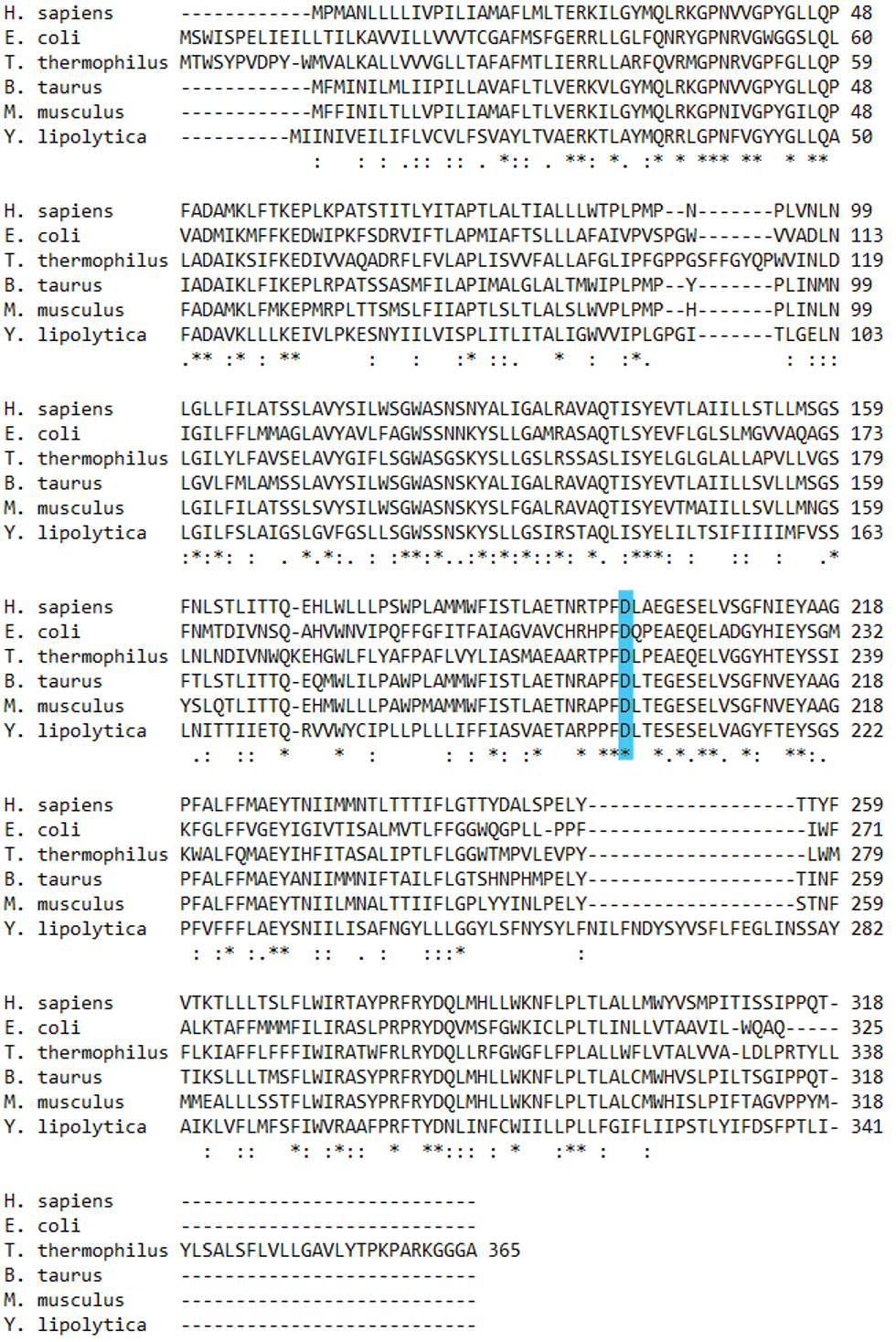

Supplement: Supplementary file 1 [file Image_1.tif]
